# Epigenetic patient stratification via contrastive machine learning refines hallmark biomarkers in minoritized children with asthma

**DOI:** 10.21203/rs.3.rs-5066762/v1

**Published:** 2024-09-13

**Authors:** Aditya Gorla, Jonathan Witonsky, Jennifer R. Elhawary, Zeyuan Johnson Chen, Joel Mefford, Javier Perez-Garcia, Scott Huntsman, Donglei Hu, Celeste Eng, Prescott G. Woodruff, Sriram Sankararaman, Elad Ziv, Jonathan Flint, Noah Zaitlen, Esteban Burchard, Elior Rahmani

**Affiliations:** 1 Bioinformatics Interdepartmental Program, University of California Los Angeles, Los Angeles, CA, USA; 2 Division of Allergy, Immunology, and Bone Marrow Transplant, Department of Pediatrics, University of California San Francisco, San Francisco, CA, USA; 3 Department of Medicine, University of California, San Francisco, San Francisco, CA, USA; 4 Department of Computer Science, University of California Los Angeles, Los Angeles, CA, USA; 5 Department of Neurology, University of California Los Angeles, Los Angeles, CA, USA; 6 Genomics and Health Group, Department of Biochemistry, Microbiology, Cell Biology, and Genetics, University of La Laguna, La Laguna, Spain; 7 Department of Computational Medicine, David Geffen School of Medicine, University of California Los Angeles, Los Angeles, CA, USA; 8 Department of Human Genetics, University of California Los Angeles, Los Angeles, CA, USA; 9 Department of Psychiatry and Behavioral Sciences, Brain Research Institute, University of California Los Angeles, Los Angeles, CA, USA; 10 Department of Neurology, David Geffen School of Medicine, University of California Los Angeles, Los Angeles, CA, USA; 11 Department of Bioengineering and Therapeutic Sciences, University of California San Francisco, San Francisco, CA, USA

## Abstract

Identifying and refining clinically significant patient stratification is a critical step toward realizing the promise of precision medicine in asthma. Several peripheral blood hallmarks, including total peripheral blood eosinophil count (BEC) and immunoglobulin E (IgE) levels, are routinely used in asthma clinical practice for endotype classification and predicting response to state-of-the-art targeted biologic drugs. However, these biomarkers appear ineffective in predicting treatment outcomes in some patients, and they differ in distribution between racially and ethnically diverse populations, potentially compromising medical care and hindering health equity due to biases in drug eligibility. Here, we propose constructing an unbiased patient stratification score based on DNA methylation (DNAm) and utilizing it to refine the efficacy of hallmark biomarkers for predicting drug response. We developed Phenotype Aware Component Analysis (PACA), a novel contrastive machine-learning method for learning combinations of DNAm sites reflecting biomedically meaningful patient stratifications. Leveraging whole-blood DNAm from Latino (discovery; n=1,016) and African American (replication; n=756) pediatric asthma case-control cohorts, we applied PACA to refine the prediction of bronchodilator response (BDR) to the short-acting β2-agonist albuterol, the most used drug to treat acute bronchospasm worldwide. While BEC and IgE correlate with BDR in the general patient population, our PACA-derived DNAm score renders these biomarkers predictive of drug response only in patients with high DNAm scores. BEC correlates with BDR in patients with upper-quartile DNAm scores (OR 1.12; 95% CI [1.04, 1.22]; P=7.9 e-4) but not in patients with lower-quartile scores (OR 1.05; 95% CI [0.95, 1.17]; P=0.21); and IgE correlates with BDR in above-median (OR for response 1.42; 95% CI [1.24, 1.63]; P=3.9e-7) but not in below-median patients (OR 1.05; 95% CI [0.92, 1.2]; P=0.57). These results hold within the commonly recognized type 2 (T2)-high asthma endotype but not in T2-low patients, suggesting that our DNAm score primarily represents an unknown variation of T2 asthma. Among T2-high patients with high DNAm scores, elevated BEC or IgE also corresponds to baseline clinical presentation that is known to benefit more from biologic treatment, including higher exacerbation scores, higher allergen sensitization, lower BMI, more recent oral corticosteroids prescription, and lower lung function. Our findings suggest that BEC and IgE, the traditional asthma biomarkers of T2-high asthma, are poor biomarkers for millions worldwide. Revisiting existing drug eligibility criteria relying on these biomarkers in asthma medical care may enhance precision and equity in treatment.

## Introduction

Asthma is the most common chronic condition in childhood^1^ and is considered a heterogeneous disease with subtypes that respond differently to therapies^2^. Identification of biomedically meaningful disease subtypes is, therefore, a critical step toward realizing the promise of precision medicine for asthma. Clinical heterogeneity may reflect variation in disease mechanisms across individuals due to genetics^3^, environmental exposures^4^, and the interaction between the two^5^. Multiple asthma endotypes with various underlying molecular mechanisms have been identified and linked to disease severity and clinical trajectory^6,7^. However, asthma treatment response remains heterogeneous, and disease control remains suboptimal for many patients^6,8^.

Different asthma endotypes, such as type 2 (T2)-high and T2-low asthma, exhibit distinct pathophysiological mechanisms and inflammatory profiles, each associated with specific biomarkers that guide clinical decision-making^9^. For example, the T2-high asthma endotype, driven by type 2 effector cells (type 2 helper T, cytotoxic T, and innate lymphoid cells) that secrete interleukin (IL)-4, IL-5, and IL-13, is primarily characterized by eosinophilic inflammation, which is often accompanied by an elevated peripheral blood eosinophil count (BEC)^10^. In contrast, T2-low asthma lacks systemic eosinophilia and is, instead, characterized by neutrophilic or paucigranulocytic inflammation and is often linked to severe, treatment-resistant forms of the disease^11^. Identifying a specific endotype by analyzing biomarkers such as BEC, sputum eosinophil percentage, fraction of exhaled nitric oxide (FeNO), and total serum immunoglobulin E (IgE) level allows clinicians to develop personalized treatment strategies^12^.

The provision of insurance coverage for most of the advanced, yet costly, targeted biologic therapies—approximately $40,000 annually^13^—relies on the presence of specific biomarkers associated with T2-high asthma^14^. However, clinical outcomes of biologic treatment vary among patients, and these biomarkers appear clinically imprecise in predicting treatment outcomes in some patients. For instance, while biologic therapy can achieve clinical remission, a study of severe asthma in adult patients reported remission in less than a quarter of the cases^15^; and in pediatric patients with elevated BEC and IgE, treatment with the IL-4 receptor antagonist Dupilumab only halves the risk of exacerbations compared to a placebo over a year^2^, suggesting that its efficacy may be limited in certain subgroups of the targeted population.

Unrecognized clinical heterogeneity within existing endotype definitions will limit the precision of known biomarkers. Differences in clinical outcomes are evident across patient groups, including racially and ethnically diverse populations. For instance, African American and Puerto Rican children exhibit a reduced bronchodilator response (BDR) to short-acting β2-agonists (SABAs)^16^, the standard treatment for acute bronchospasm, even when combined with inhaled corticosteroids (ICS)^17^. Together with variability in biomarker distribution among diverse populations^18^, asthma clinical heterogeneity can compromise medical care and hinder health equity due to biases in drug eligibility^19^.

Practical, robust, and unbiased patient stratification will advance precision and equity in medical care for the large population of patients with asthma. Genomics may offer an inherently unbiased view of disease mechanisms^3,20^, enabling data-driven approaches to more accurately capture asthma heterogeneity^21,22^. Here, we propose a novel contrastive machine learning method we developed to define a patient stratification score based on patterns of DNA methylation (DNAm) from whole blood, which has been linked to asthma pathogenesis and treatment response^23^. Our DNAm score reveals a continuum of variation within the T2 asthma endotype, along which the traditional asthma biomarkers BEC and IgE are predictive of drug response only among patients with high DNAm scores.

## Results

### A novel contrastive learning method for learning disease heterogeneity

Current data-driven disease subtyping efforts predominantly rely on data clustering and latent representation techniques, ranging from classical unsupervised methods like K-means^24^ and principal component analysis^25^ to deep-learning approaches such as autoencoders^26^. These methods, whether explicitly or implicitly, rely on data-driven similarities between samples, which can obscure the identification of meaningful subtypes, especially when those subtypes are reflected by subtle signals in the data. Because these techniques analyze all features in the data, without additional constraints, they tend to emphasize *dominant* variation – sources of variation that correlate with many features in the data. In genomic data, however, these dominant sources of variation typically represent unwanted factors, such as cell-type composition^27^, batch effects^28^, or ancestry^29^, rather than the true heterogeneity of the disease.

We developed Phenotype Aware Component Analysis (PACA), a novel contrastive machine-learning method for defining biomedically meaningful disease subtypes and patient stratifications from high-dimensional data (Supplementary Fig. 1a). Unlike standard approaches, PACA does not simply cluster patients based on the dominant variation in the data. Instead, PACA isolates disease-specific heterogeneity by first removing sources of variations that are shared with healthy control individuals from the disease case data, and then applying standard dimensionality reduction techniques (Supplementary Fig. 1a). By accounting for sources of variation present in both cases and controls, we expect the top axes of variation in case data to reflect disease heterogeneity. Since these shared sources of variation are generally unknown, we take an unsupervised approach to identify latent features that are consistent across the two groups. See Methods and Supplementary Note S1 for more details.

We confirmed that PACA is statistically calibrated and better powered to identify disease heterogeneity compared to other contrastive methods^30,31^ when applied to synthetic and real-world data, including gene expression, DNAm, and genotype data (Supplementary Fig. S1b and S2-S5; Supplementary Note S2). Unlike PACA, existing contrastive learning methods^30,31^ require tuning a vague contrastive hyperparameter, which controls the level of variation among cases compared to controls. Such hyperparameters are likely to be misspecified when seeking to learn unknown disease heterogeneity (Supplementary Note S3), often leading to power loss or the false tagging of arbitrary patterns as real disease heterogeneity (Supplementary Fig. S6).

### An epigenetic patient stratification score associates with drug response in pediatric asthma cohorts

We utilized PACA to develop an asthma patient stratification score using whole-blood DNAm from the Genes-environments & Admixture in Latino Americans II (GALA II), a pediatric asthma cohort primarily of Mexican and Puerto Rican ethnicities (n=618 cases, n=398 controls; Supplementary Table 1). To reduce noise and narrow the feature space in the data, we first evaluated all methylation sites (>730,000 cytosine-phosphate-guanine probes, or CpGs) for statistical association with asthma (see Methods). An analysis with PACA considering only CpGs that demonstrated a nominally significant association with asthma (P<0.01) resulted in a patient stratification model based on a linear combination of 7,662 CpGs. To confirm the cross-population consistency of the resulting asthma stratification, we applied the DNAm score to a pediatric African American patient replication cohort with whole-blood DNAm from the Study of African Americans, Asthma, Genes, and Environments (SAGE II; n=429 cases; Supplementary Table 1).

**Fig. 1:**
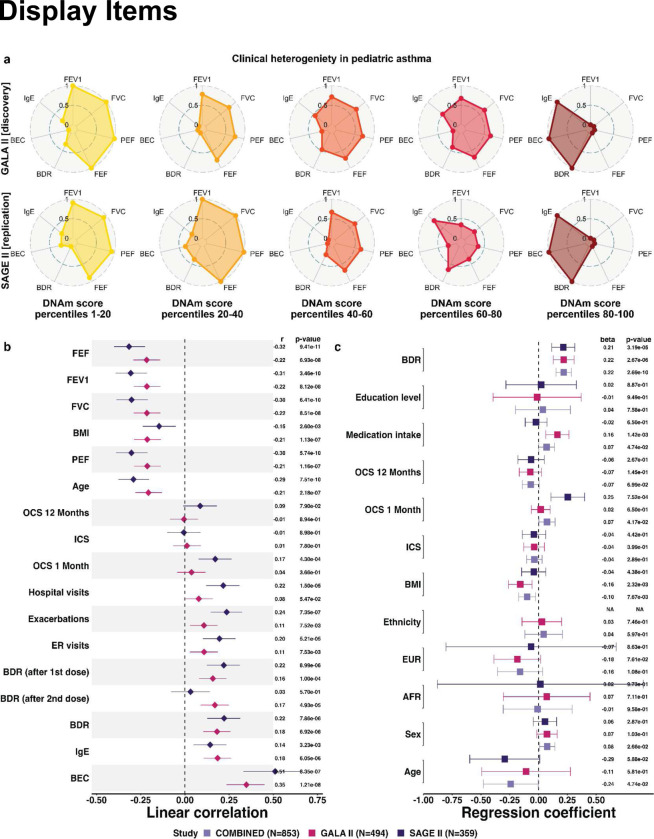
PACA DNAm scores stratify patients along a continuum corresponding to heterogeneity in the clinical presentation of asthma. (a) Radar plots illustrating distribution shifts of asthma phenotypes across different percentile ranges of the DNAm score. Each point on the radial scale represents an average phenotypic value for the respective quantile range, normalized on a 0 to 1 scale within each cohort. (b) Pearson’s coefficients and 95% confidence intervals (CIs) for the correlation between the DNAm scores and clinical covariates. (c) Linear regression coefficients and 95% CIs for the DNAm score as the outcome and BDR as the variable of interest, adjusted for demographic and clinical variables. Results are stratified by study (GALA II, SAGE II) or combined (COMBINED).

In both GALA II and SAGE II, the DNAm score stratified patients along a continuum corresponding to heterogeneity in lung function and clinical presentation of asthma (Fig. 1a,b). Specifically, high DNAm scores are associated (Pearson correlation; Bonferroni adjusted P<0.05) with lower baseline lung function as measured by forced vital capacity (FVC), peak expiratory flow rate (PEF), forced expiratory volume in one second (FEV1), and forced expiratory flow (FEF). Other asthma phenotypes associated with high DNAm scores include higher BDR, elevated BEC and IgE, and higher exacerbation scores (Methods; Fig. 1b; Supplementary Tables S2 and S3).

The associations with BDR, BEC, and IgE remained significant (linear regression; GALA II P<2.7e-6, SAGE II P<2.9e-3) after adjusting for demographics (age, sex, ancestry, and ethnicity), body mass index (BMI), education level, medication intake, and inhaled (ICS) and oral (OCS) corticosteroids use (Fig. 1c; Supplementary Fig. S7). Inspecting the top 20 most informative CpGs of our stratification model revealed heavily weighted CpGs in genes implicated in asthma pathogenesis and regulation of airway inflammation and remodeling (*STAT3*, *RASSF1*, *MEOX1*)^32–34^, bronchodilator response (*DDX54*)^35^, and asthma severity and lung function (*ALDH2*)^36,37^ (Supplementary Tables S5 and S6). We confirmed that existing contrastive learning methods did not yield similarly meaningful asthma patient stratification (Supplementary Table S2 and S4; Supplementary Note S2.7).

### BEC and IgE predict drug response only in patients with high DNAm scores

We evaluated whether our patient stratification model can explain the heterogeneity of biomarkers in predicting clinical outcomes, focusing on BDR to the short-acting β2-agonist albuterol, the most used drug for acute bronchospasm worldwide. The widely used asthma biomarkers BEC and IgE, both found to be associated with our DNAm score (Fig. 1), are known to be predictive of BDR^38^. Across GALA II and SAGE II, the odds ratio (OR) for drug response (BDR≥12%) is 1.14 (95% CI [1.1, 1.18]) and 1.23 (95% CI [1.12, 1.35]) for BEC and IgE, respectively (Supplementary Fig. S8).

We next investigated if the epigenetic score stratifies the population such that these biomarkers are predictive of BDR only in specific patient subgroups along the stratification spectrum (i.e., a statistical interaction). A linear regression model for BDR as the outcome revealed a significant interaction between IgE levels and the DNAm score (GALA II P=4.8e-3; SAGE II P=3.3e-6) after adjusting for IgE levels, demographics, BMI, education level, medication intake, ICS, and OCS (Supplementary Fig. S9). Similarly, we found a significant interaction between BEC and the DNAm score in GALA II (P=5.2e-5; Supplementary Fig. S9). This result was not replicated in SAGE II, likely owing to the limited number of BEC measurements available in this cohort (n=83). Even so, imputing missing BEC proportions from DNAm levels^39^ (Supplementary Fig. S10) revealed a significant interaction between BEC and our DNAm score, which could not be explained by the BEC composition itself (GALA II P=8.3e-3, SAGE II P=4.4e-4; Supplementary Fig. S9).

**Fig. 2:**
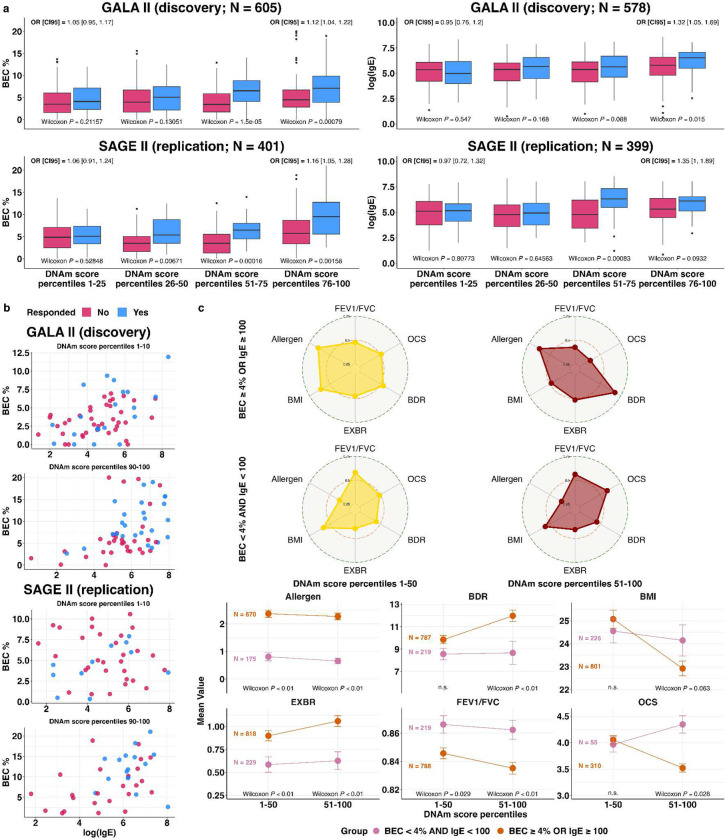
BEC and IgE as drug response biomarkers along the DNAm patient stratification spectrum. (a) Grouped boxplots comparing the distributions of imputed blood eosinophil proportions (BEC%; left) and (log) IgE levels (right) across different percentile ranges of the DNAm score, stratified by BDR responders and non-responders. Low Wilcoxon rank-sum p-values and high odds ratio (with 95% confidence intervals) indicate the biomarker is predictive of BDR for patients in the respective percentile range. (b) The empirical joint distribution of BEC% and (log) IgE in patients with top and bottom decile DNAm scores, stratified by BDR responders and non-responders. (c) Distribution shifts in baseline presentation of clinical phenotypes known to be associated with response to anti-T2 asthma biologic drugs. Results are presented across both GALA II and SAGE II for better statistical power. Error bars in line plots indicate the standard error of the mean (SEM). Low Wilcoxon rank-sum p-values indicate differences in phenotype presentation between patient groups in the respective percentile range of the DNAm score. EXBR: Exacerbation score in the last 12 months (0–6); Allergen: Number of positive reactions on a skin prick allergen test; OCS: Time since the last oral steroid prescription (a lower value indicates a more recent prescription).

The statistical interactions suggest that elevated BEC and IgE predict BDR only in patients with high DNAm scores. In GALA II, classifying patients into responders (BDR≥12%) and non-responders, IgE levels correlate with drug response in patients with above-median methylation scores (OR for response 1.42; 95% CI [1.24, 1.63]; P=3.9e-7) but not in patients with below-median scores (OR 1.05; 95% CI [0.92, 1.2]; P=0.57); (imputed) BEC correlates with response in upper-quartile (OR 1.12; 95% CI [1.04, 1.22]; P=7.9 e-4) but not in lower-quartile patients (OR 1.05; 95% CI [0.95, 1.17]; P=0.21) (Fig. 2). More generally, we observe a linear increase in the correlation of BEC and IgE with response along the DNAm score continuum. These results were replicated in SAGE II (Fig. 2); results combining both datasets are provided in Supplementary Fig. S11. Since low baseline lung function may explain BDR^38^, we further verified that our results hold when restricting the analysis to patients with low baseline lung function (Supplementary Fig. S12; Methods). Overall, our results confirm a robust monotonous increase in the effectiveness of elevated BEC and IgE as predictive drug response biomarkers along the patient stratification spectrum.

Next, we evaluated the performance of logistic regression models using both BEC and IgE as two-biomarker models for BDR prediction (Supplementary Table S7). A standard two-biomarker model based on the entire patient population yielded ROC AUC 0.62 and 0.69 on GALA II (train) and SAGE II (test), respectively. In contrast, a two-biomarker model for patients with top-decile DNAm scores achieved ROC AUC of 0.76 and 0.75 in GALA II and SAGE II, respectively. As expected, a two-biomarker model for patients with bottom-decile DNAm scores, for which BEC and IgE are not associated with BDR, performed poorly (ROC AUC of 0.62 and 0.59). Evaluating other percentile ranges demonstrated a linear increase in performance along the DNAm score continuum (Supplementary Table S7).

We further evaluated alternative predictive models for BDR. A logistic regression model using cell-type composition, a significant source of variation in DNAm data^40^, underperformed compared to the two-biomarker models tailored for patients with high DNAm scores (Supplementary Table S7). A DNAm-based regularized logistic regression model using the same 7,662 CpGs that define our DNAm score outperformed the two-biomarker models in predicting BDR in GALA II (ROC AUC 0.83). However, unlike the simple two-biomarker model for patients with high DNAm scores, the performance of the DNAm-based predictive model did not replicate in the SAGE II cohort (ROC AUC 0.65). The likely overfitting to confounding effects, presumably leading to this poor consistency across cohorts, underscores PACA’s effectiveness in eliminating unknown confounding factors. This is achieved by using contrastive learning to remove sources of variation that are shared across cases and controls. Interestingly, both the cell-type composition and DNAm-based regularized logistic regression models performed better when evaluated in patient groups with high DNAm scores, further underscoring the robustness of the proposed DNAm score in stratifying clinical outcomes (Supplementary Table S7).

### The DNAm patient stratification score reflects heterogeneity of T2 asthma

The association of high DNAm scores with T2-high asthma (elevated BEC and IgE; Fig. 1b) could suggest that our DNAm score simply recapitulates known T2 endotypes. However, the DNAm score stratified the effectiveness of BEC and IgE in predicting BDR even when restricting the analysis only to T2-high patients, as defined by BEC-high or IgE-high levels (Supplementary Fig. S12; Methods). In contrast, this was not the case when considering only T2-low patients (Supplementary Fig. S13), suggesting that our patient stratification primarily represents unknown variation that pertains to T2 asthma.

Most existing biologic treatments for asthma target the T2 pathway. Given this, we explored whether elevated BEC and IgE levels might be more suggestive of response to biologic drugs in patients with a high DNAm score, similar to their predictive value for BDR. We stratified patients based on their BEC and IgE levels, which are primary biomarkers of response to T2-targeted biologic drugs. We first observed that the DNAm score is associated with a difference in clinical presentation only among high-BEC or high-IgE patients, providing further evidence that our DNAm score reflects a spectrum of variation within the T2-high asthma endotype (Fig. 2c). Second, among patients with high DNAm scores, we observed that elevated BEC or IgE is associated with baseline clinical presentation that is known to benefit more from biologic treatment, including higher exacerbation scores^41,42^, higher allergen sensitization^43^, lower BMI^44,45^, more recent OCS prescription^45,46^, and lower FEV1/FVC ratio^41^. In contrast, these characteristics are not as marked among patients with low DNAm scores, providing strong evidence that BEC and IgE are better predictors of biologic drug response among patients with high DNAm scores.

Finally, we asked whether epigenetic modifications specific to T2-high asthma drive our DNAm-based patient stratification and whether such modifications can be attributed to certain cell types. Using a deconvolution method for bulk DNAm^47^, we tested our model’s top 20 most informative CpGs for cell-type-level differential DNAm with the DNAm scores while accounting for demographics, technical variation, and cell-type composition. We identified three CpGs associated with the DNAm score in eosinophil cells: cg18399629, cg20263733, and cg14617280 (Supplementary Table S8). These CpGs, identified in GALA II (Bonferroni adjusted P<0.05) and replicated in SAGE II (P<0.012), present hypermethylation in eosinophil cells of samples with high DNAm scores; other cell types do not present differential methylation in these CpGs. Breaking the analysis into T2-high and T2-low patient groups (Methods) showed the statistical signal is driven by the T2-high patients (Supplementary Table S9 and S10), suggesting that eosinophils-specific differential epigenetic programming may explain the variation within T2 asthma identified by our DNAm stratification model.

## Discussion

Our findings indicate that DNAm scores have the potential to enhance the clinical utility of established biomarkers and identify appropriate patient subgroups, leading to more personalized asthma management strategies. Among patients with low DNAm scores, the traditional asthma biomarkers BEC and IgE are ineffective for predicting BDR and may also be less useful for identifying those who would benefit from other therapies targeting T2-high asthma. Embracing a molecular approach based on epigenetics that integrates genetic and environmental factors, our DNAm-based asthma stratification score proves robust across populations and patient groups defined by established asthma phenotypes.

The DNAm score moves away from categorizing childhood asthma into discrete phenotypic or endotype clusters, instead highlighting that treatment response and biomarker effectiveness can exist along a predictable continuum. This approach aligns with the growing call for molecular profiles that define asthma phenotypes and endotypes more precisely, allowing for targeted “personalized” therapies^48^. Our findings suggest that asthma heterogeneity, particularly within the T2-high endotype, is more complex than previously recognized.

Clinical responses to T2-targeting biologics for asthma vary, even among patients with similar eligibility criteria^2,15^, highlighting the influence of multiple factors on asthma manifestation and treatment response. The apparent complexity of the T2-high endotype highlights the potential for identifying novel sub-phenotypes and developing more tailored treatment strategies. In particular, our results serve as a proof-of-principle for contrastive learning and epigenetics to robustly identify responders and possibly “super-responders”^42,45^ to anti-T2 biological agents among children with suboptimal responses to therapy, regardless of their perceived endotype status.

Integrating whole-blood epigenome-wide DNA methylation data with our novel contrastive machine learning algorithm may enhance our understanding of the complex pathophysiological processes underlying asthma and help improve treatment strategies beyond response to bronchodilators. The association of reversible airway obstruction with eosinophilic inflammation^38^, a hallmark of T2 asthma, suggests a shared pathophysiology, indicating that patients who respond to albuterol will also respond well to biologics. While our data does not include direct measures of biologic response, we observe that samples with high DNAm scores are enriched with eosinophil cells exhibiting hypermethylated sites near genes implicated in T2-high asthma and biologic drug response, including *CREG1* and *MIR4765*^49,50^. The conjectured epigenetically encoded memory in these eosinophil cells, as reflected by our DNAm score, may therefore align with the mechanisms targeted by other asthma therapeutics^51^. Furthermore, combining BEC and IgE with our DNAm score allowed us to identify a group of patients with a clinical profile known to respond to these drugs^15,41,42,44–46^. However, further studies with larger sample sizes and a broader range of drugs, along with corresponding response data, are necessary to confirm the utility of this score and to establish its potential to enhance prospective clinical trials and inform therapeutic recommendations.

## Methods

### Study populations

Clinical, biomarker, spirometric, and DNA methylation (DNAm) data were collected as part of GALA II and SAGE II, two case-control studies of asthma conducted between 2006 and 2014. GALA II enrolled participants from five urban study centers across the United States (Chicago, Bronx, Houston, San Francisco Bay Area, and Puerto Rico), while SAGE II recruited participants exclusively from the San Francisco Bay Area. Participants, self-identifying as African American with four African American grandparents in SAGE II and as Latinos in GALA II, ranged from 8 to 21 years at recruitment. Case participants were defined by physician-diagnosed asthma, presence of asthma symptoms, or use of asthma medication within the last 2 years, excluding any history of other lung or chronic nonallergic illnesses. Healthy control participants exhibited no lifetime history of asthma or allergies and reported no instances of coughing, wheezing, or shortness of breath in the two years preceding enrollment. Case and control participants were matched at a 1:1 ratio based on age within one year at each recruitment site. Exclusion criteria comprised individuals in their third trimester of pregnancy, current smokers, and those with a smoking history of at least 10 pack-years. The University of California San Francisco Human Research Protection Program Institutional Review Board approved study protocols for GALA II and SAGE II.

### Epigenome-wide methylation assessment and quality control

Samples for DNAm assessment were collected at the same time when lung function and BDR were measured. Genomic DNA was extracted from whole blood using Wizard Genomic DNA Purification Kits (Promega, Fitchburg, WI). DNAm measurements were obtained at more than 850,000 5’-cytosine-phosphate-guanine-3’ dinucleotide (CpG) sites from whole blood using the Infinium Illumina MethylationEPIC array (Illumina, San Diego, Calif). Quality control was conducted using the ENmix R package (version 1.22.0), involving background and dye bias correction, probe-type bias adjustment, inter-array normalization, and the removal of low-quality CpG probes and samples [1]. We excluded probes that are either polymorphic or cross-reactive based on the reference panel created by McCartney et al. [2, 3]. Methylation intensities were used to calculate beta values, and outlier or missing DNAm data points were replaced by imputed values unless the probe or sample missingness rate exceeded 5% and 10%, respectively. See additional pre-processing information in Supplementary Note S4.1.

### Biomarkers, Spirometric, and BDR Assessments

Serum total IgE levels were measured twice using the Phadia 100 system (ThermoFisher Scientific in Uppsala, Sweden). If the results were not within 10% agreement, a third measurement was taken. We applied log transformation to the serum total IgE values to address the right-skewed distribution of the data on its natural scale. Peripheral BECs were obtained from complete blood cell counts (CBC) with differentials from certified laboratories. GALA II used Quest Diagnostics while the University of California, San Francisco Clinical Laboratories was used for SAGE II.

Spirometric measurements were obtained from all participants both before and 15 minutes after the administration of four puffs of albuterol (90 *μ*g per puff), following the American Thoracic Society recommendations [4]. Spirometry was conducted for a third time following a second albuterol dosage (two puffs for those <16 years old or four puffs for those ≥16 years old). Spirometry and bronchodilator testing were conducted using a KoKo^®^ PFT Spirometer (nSpire Health Inc., Louisville, CO). All asthma medications were held for 12 hours prior to the spirometry assessment. Pulmonary function percent predicted (observed×100/predicted) baseline and z-scores were calculated using the Global Lung Function Initiative race-neutral equations [5]. BDR was determined by assessing the percentage change in measured forced expiratory volume in one second (FEV1) before and after albuterol administration, utilizing post-albuterol spirometry with the greatest observed change. Positive BDR (response) was defined as an increase in FEV1 from baseline equal to or exceeding 12% predicted. BDR was evaluated concurrently with the collection of whole-blood samples used for collecting blood biomarkers and DNAm profiling.

### Imputing missing blood eosinophil counts

BEC and CBC measurements are only available for 255 (37.2%) of the GALA II and 83 (19.3%) of the SAGE II cases. To impute eosinophil proportions for samples with missing BEC levels, we applied the cell-type decomposition approach described in the method BayesCCE [6] (“impute mode”) as follows. First, we leveraged the available CBC measurements from a subset of the GALA II cases and the corresponding whole-blood DNAm profiles of these samples to estimate cell-type specific DNAm signature profiles. We considered cell-type signatures of 450 CpGs previously identified to be informative for leukocyte mixture decomposition [7]. Samples with abnormal measurements, defined as those with more than 2% deviation between their whole-blood counts (WBC) and the sum of their neutrophil, eosinophil, lymphocyte, monocyte, and basophil absolute counts, or those with reported eosinophil cell-type proportions exceeding 30%, were excluded from the step of estimating signature profiles. Given cell-type DNAm profiles estimated from the known cell compositions, we imputed missing cell-type proportions for participants from both cohorts using quadratic programming [6].

### Covariates and asthma phenotypes

In all linear regression models, including the interaction models, we accounted for age, sex, body mass index (BMI), fractions of African and European genetic ancestry (Supplementary Note S4.4), Mexican ethnicity (indicator; self-reported), education level (on an ordinal scale; self-reported), medication use frequency in the last 2 weeks (self-reported), inhaled corticosteroid (ICS) use (self-reported), and oral corticosteroids (OCS) use in the last month and year (self-reported). In any analysis combining both GALA II and SAGE II, we also accounted for the cohort (a binary indicator variable). In any given analysis, we considered only samples with no missing covariates or phenotypes. Further information, including the description of asthma phenotypes, including exacerbation count, hospital visits, emergency room visits in the last year, and the number of positive skin prick allergen sensitizations, is provided in Supplementary Notes S4.2–4.5.

### PACA: Phenotype Aware Component Analysis

#### Problem setup

We consider a low-rank model for high-dimensional data coming from a target population and a background population. Both populations share the same biological and non-biological sources of variation, with the exception of an additional component in the target population that represents phenotypic heterogeneity. Hereafter, we refer to the target and background groups as “cases” and “controls”, respectively.

Let $X\in R^{m\times n_{1}}$ be a matrix of measurements of m features for $n_{1}$ cases, and let $Y\in R^{m\times n_{0}}$ be a matrix of measurements of the same m features for $n_{0}$ control individuals, such that $m>max\left(n_{0},n_{1}\right)$. We consider the following descriptive model:

(1) $$X=W^{0}Z_{X}^{0}+W^{1}Z_{X}^{1}+E_{X},\;{\;\;\left(E_{X}\right)}_{i}\sim N\left(0,\sigma^{2}I_{m}\right)$$

(2) $$Y=W^{0}Z_{Y}^{0}+E_{Y},\;\;\;{\left(E_{Y}\right)}_{i}\sim N\left(0,\sigma^{2}I_{m}\right)$$

$W^{0}\in R^{m\times k_{0}}$ denotes the sources of variation (directions in the features space $R^{m}$) of a $k_{0}$-dimensional low-rank signal (typically $k_{0}<<min\left(n_{0},n_{1}\right)$) and $Z_{X}^{0}\in R^{k_{0}\times n_{1}},$ $Z_{Y}^{0}\in R^{k_{0}\times n_{0}}$ denote components (in the sample spaces $R^{n_{0}},$ $R^{n_{1}}$) of individual-level variation for the individuals in X, Y, respectively. Altogether, these directions and components represent the shared variation across cases and controls. $W^{1}\in R^{m\times k_{1}}$ and $Z_{X}^{1}\in R^{k_{1}\times n_{1}}$ denote the sources of variation of an additional $k_{1}$-dimensional low-rank signal and its corresponding individual-level component, representing the phenotypic heterogeneity of the cases in $X.\;Z_{X}^{1}$, by construction, represents variation specific to cases and is where we expect to find variation in disease. Therefore, our goal is to learn $Z_{X}^{1}$ (up to a linear transformation). Of note, $Z_{X}^{1}$ may be more subtle and explain much less of the variation in the data compared to $Z_{X}^{0}$.

#### Orthogonality assumption

We make the assumption that $W^{0}⊥W^{1}$. That is, we assume the case-specific sources of variation are orthogonal to the axes of common variation across cases and controls. We refer to this assumption as the orthogonality assumption. Neglecting this assumption, while not incorporating further supervision, may lead to under-correction for shared sources of variation across cases and controls. This, in turn, can lead to falsely tagging background variation as phenotypic heterogeneity (Supplementary Fig. S6).

Under Eq. (1)–(2) and the orthogonality assumption, learning case-specific variation amounts to finding sources of variation in X that do not exist in Y. However, one should not necessarily remove from X all the variation shared across cases and controls for identifying case-specific variation, as this may be suboptimal. For instance, in cPCA, a contrastive method similar in spirit to PACA, incorporating the orthogonality assumption by setting a very large contrastive hyperparamter can be shown to be suboptimal (Supplementary Note S3). An arguably better approach is to condition only on sources of variation that exist in both X, Y and are more dominant in the data compared to the case-specific variation. Such sources of variation are expected to be of much lower dimension than the dimension of the observed data. This is exactly the key idea behind PACA.

#### The PACA algorithm

Using a singular value decomposition we can consider an alternative formulation for the model in (1)–(2):

(3) $$X=U^{0}\Sigma_{X}^{0}{\left(V_{X}^{0}\right)}^{⊤}+U^{1}\Sigma_{X}^{1}{\left(V_{X}^{1}\right)}^{⊤}+E_{X}\;\text{s.t.}\;U^{0}⊥U^{1}$$

(4) $$Y=U^{0}\Sigma_{Y}^{0}{\left(V_{Y}^{0}\right)}^{⊤}+E_{Y}$$

where each of $U^{0}\in R^{m\times k_{0}},$ $V_{X}^{0}\in R^{n_{1}\times k_{0}},$ $V_{Y}^{0}\in R^{n_{0}\times k_{0}},$ $U^{1}\in R^{m\times k_{1}}$, and $V_{X}^{1}\in R^{n_{1}\times k_{1}}$ represents an orthonormal basis. Under this presentation, we are interested in learning $V_{X}^{1}$, a (possibly linearly-transformed) surrogate for $Z_{X}^{1}$ in Eq. (1). Given the directions of the shared sources of variation $U^{0}$, note that

(5) $$E\left[U^{0}{\left(U^{0}\right)}^{⊤}X\right]=U^{0}\Sigma_{X}^{0}{\left(V_{X}^{0}\right)}^{⊤}$$

which establishes a way to remove the expected effect it induces on X (i.e., the signals in X that are coming from sources of variation that exist in both cases and controls). Our PACA algorithm, therefore, estimates and removes the effects of $U^{0}$ on X, followed by PCA for capturing $V_{X}^{1}$. Our main focus is thus estimating $U^{0}$.

Since $U^{0}$ is found in both X, Y, we can estimate it by employing a canonical correlation analysis (CCA) [8]. Importantly, unlike in a typical application of CCA, where we wish to find linear transformations of the features that yield the highest correlation between the same set of samples in two datasets (i.e., vectors in the samples space), here, we seek linear transformations of the samples that provide vectors in the features space $R^{m}$. Specifically, we find the first pair of canonical variables by solving:

(6) $$\overset{ˆ}{a},\overset{ˆ}{b}=\underset{a\in R^{n_{1}},b\in R^{n_{0}}}{argmax}a^{⊤}X^{⊤}Yb\text{,}\;\text{s.t.}∥Xa{∥}_{2}=1,∥Yb{∥}_{2}=1$$

Setting ${\overset{ˆ}{u}}_{1}^{0}=X\overset{ˆ}{a}$ yields the representation in X of the strongest direction of shared variation across X and Y. Let $S_{XY},$ $S_{XX}$ and $S_{YY}$, be the empirical sample cross-covariance and sample covariance matrices of X and Y. The solution for $\overset{ˆ}{a},$ $\overset{ˆ}{b}$ is known to be given by the eigenvector that corresponds to the top eigenvalue of $S_{XX}^{-1}S_{XY}S_{YY}^{-1}S_{YX}$ and the eigenvector that corresponds to the top eigenvalue of $S_{YY}^{-1}S_{YX}S_{XX}^{-1}S_{XY}$, respectively [8, 9]. This procedure can be repeated iteratively by restricting the vectors ${\overset{ˆ}{u}}_{r}^{0}$ to be orthogonal to the previous canonical variables ${\overset{ˆ}{u}}_{1}^{0},\ldots ,{\overset{ˆ}{u}}_{r-1}^{0}$ (and similarly for the variables of Y). Eventually, the collective of these vectors ${\overset{ˆ}{U}}^{0}$ can be used for removing the shared sources of variation from X following Eq (5); see a summary of PACA in Algorithm 1.

The number of canonical directions representing sources of variation shared between cases and controls is unknown. Accounting for too few canonical directions would result in PACA capturing variation unrelated to the heterogeneity of the condition of interest. Accounting for too many canonical directions, on the other hand, is expected to lead to power loss. We, therefore, employ a permutation scheme to identify the minimal number of canonical directions (k) that need to be removed to identify significant case-specific variation (Supplementary Algorithm S.A.1; Supplementary Note S1.1).

Lastly, PACA is constrained by the same limitations as a standard CCA, which requires in our setup more features than samples. To address scenarios where this requirement is unmet, we developed a randomized extension of PACA (rPACA), which operates in the regime of more samples than features by estimating the PACA components via random data sampling (Supplementary Algorithm S.A.2; Supplementary Note S1.2).

**Algorithm 1:** Phenotype Aware Components Analysis (PACA)

**Input:** case data $X\in R^{m\times n_{1}}$, control data $Y\in R^{m\times n_{0}}$, shared dimensions to remove k,m: shared features, $n_{1}$: case sample size, $n_{0}$ control samples size
1: $X_{ij}^{\prime }\leftarrow X_{ij}-\frac{1}{m}\sum_{l=1}^{m}\;X_{lj},$ $Y_{ij}^{\prime }\leftarrow Y_{ij}-\frac{1}{m}\sum_{l=1}^{m}\;Y_{lj}$ ⊳ Mean centering the matrices
2: **for** r←1,…,k **do** ⊳ Core CCA algorithm
${\overset{ˆ}{a}}_{r},{\overset{ˆ}{b}}_{r}=\;\underset{a_{r}\in R^{n_{1},b_{r}\in R^{n_{0}}}}{argmax}a_{r}^{⊤}X^{\prime ⊤}Y^{\prime }b_{r}\;\;\text{s.t.,}\;{∥X^{\prime }a_{r}∥}_{2}=1,{∥Y^{\prime }b_{r}∥}_{2}=1\;\;\forall 1\leq h<r:X^{\prime }a_{h}⊥X^{\prime }a_{r},Y^{\prime }b_{h}⊥Y^{\prime }b_{r}$
3: $\overset{ˆ}{A}\leftarrow \left[{\overset{ˆ}{a}}_{1},\ldots ,{\overset{ˆ}{a}}_{k}\right]$
4: ${\overset{ˆ}{U}}^{0}\leftarrow X^{\prime }\overset{ˆ}{A}$
5: $\tilde{X}\leftarrow {\overset{ˆ}{X}}^{\prime }-{\overset{ˆ}{U}}^{0}{\left({\overset{ˆ}{U}}^{0}\right)}^{⊤}X^{\prime }$
6: ${\overset{ˆ}{U}}_{1},{\overset{ˆ}{V}}_{X}^{1}\leftarrow PCA({\tilde{X}}^{⊤})$ ⊳ PCA returns the PC loadings (rotations) and scores
**Output:** ${\overset{ˆ}{V}}_{X}^{1}$

### DNAm score for asthma patient stratification

To identify asthma heterogeneity in DNAm data, we first created a panel of CpGs enriched for association with asthma. We expected that proper feature selection would improve performance by reducing noise and narrowing the search space. We used TCA [10] to perform an epigenome-wide association study (EWAS) with asthma status, while accounting for age, sex, the top five genetic principal components (PCs), and the top 30 PCs calculated from the lowest variance probes (treated as control probes, as suggested elsewhere [10, 11]). Since TCA requires cell-type proportions as an input, we applied EpiDISH [12] to estimate proportions using the “cent12CT.m” reference panel [13]. This analysis resulted in a panel of 7,662 CpGs passing a nominal association threshold of p<0.01, which we retained for constructing the PACA DNAm score.

We learned the top two PACA components using the above set of 7,662 CpGs in GALA II (discovery) and applied the model to SAGE II (replication) by multiplying the coefficients of these components by the same CpGs in SAGE. To evaluate whether the PACA components capture asthma heterogeneity, we tested their linear correlation with a set of 668 phenotypes, only retaining those passing multiple-testing (Bonferroni) correction. Evaluating the consistency of these phenotype correlations across GALA II and SAGE II revealed poor consistency for the first PACA component and high consistency for the second PACA component (Supplementary Tables S2 and S3). We, therefore, set the second PACA component as the DNAm heterogeneity score.

### Stratifying the effect of BEC and IgE as biomarkers of drug response

Testing the statistical interaction of the DNAm score with BEC and IgE was based on linear regression models with BDR as the outcome. An interaction was evaluated by including in the regression a multiplicative term between the DNAm score and a given biomarker, in addition to covariates and a linear term of biomarker. Restricting this analysis to type 2 (T2)-high patients, relied on six established biomarker and phenotypic thresholds commonly used to define various T2-high asthma endotype sub-populations. Specifically, serum total IgE values were dichotomized into high and low categories using cut-offs of 100 kU/L and 200 kU/L [14]; elevated eosinophil levels as defined by peripheral BEC values based on 150 c/*μ*L and 300 c/*μ*L cut-offs [15]; and imputed BEC proportion values were binarized into high or not based on ≥ 4% cut-off [16, 17]. Testing interactions among patients with low baseline lung function was restricted to patients with a cut-off of 80% predicted percent FEV1 or a cut-off of 0.70 for the FEV1/FVC ratio [18]. Finally, reported odd ratios were calculated based on unadjusted logistic regression models with the response status (BDR≥12%) as the outcome.

### Benchmarking PACA with alternative contrastive methods

We compared PACA to standard PCA, contrastiveVAE (cVAE) [19], and contrastive PCA (cPCA) [20] with three different hyperparameter-tuning strategies. See Supplementary Note S2.3 for more details.

### Benchmarking predictive models of BDR

In all models, we used the GALA II cohort as the training set and the SAGE II cohort as the test set. Three distinct model types were assessed: a DNAm model, biomarker models, and a cell-type composition model. The DNAm model utilized the same 7,662 asthma-enriched CpGs we used in our PACA-derived DNAm score as features. These features were used in an elastic net penalized logistic regression model , trained using a 10-fold cross-validation procedure to determine the optimal lambda parameter (R’s glmnet; default α=0.5 ratio for the $\ell_{1}$ and $\ell_{2}$ penalties). For the biomarker models, we considered a logistic regression model incorporating log-transformed total IgE, imputed BEC, and their interaction as predictors; some of the models considered only subsets of the patients based on differnet percentile groups of the DNAm scores. The cell-type composition model was constructed using logistic regression with imputed fractions of lymphocytes, monocytes, basophils, neutrophils, and eosinophils as the predictors. Model performance was evaluated using the area under the receiver operating characteristic curve (AUC-ROC) and the area under the precision-recall curve (AUC-PR).

### Cell-type-level differential methylation

We used TCA [10] to identify cell-type-level differential methylation with the DNAm score. As an input to TCA, we provided the bulk DNAm levels of the top 20 most informative features of the stratification model, ranked by the absolute value of their associated coefficient. TCA models differential methylation in cell types for which proportions are available. Therefore, similarly to our application of TCA for feature selection before applying PACA, we used Epidish [12] and a methylation reference panel [13] to estimate proportions for a set of 10 immune cell types, including neutrophil, eosinophil, CD4 naïve T, CD4 memory T, CD8 naïve T, CD8 memory T, monocytes, B memory, B naïve, and natural killer cells. All hypotheses were tested in GALA II and evaluated at a significance level of 0.05 (Bonferroni adjusted); the three identified associations were replicated in SAGE.

To test for differential methylation in T2-high and T2-low patients, we used the same BEC and IgE cutoffs described earlier. Since our data include more T2-high (477 in GALA and 341 in SAGE) than T2-low (at most, 141 in GALA and 88 in SAGE) samples, for the T2-low-specific differential methylation analysis, we increased statistical power by combining both GALA II and SAGE II. TCA automatically accounts for cell-type composition using the cell-type proportions provided as input. In addition, in all models, we accounted for age, sex, the top five genetic PCs, and technical variation. For the latter, we used the top 10 PCs calculated from the lowest variance probes (treated as control probes, as suggested elsewhere [10, 11]). Of note, considering only 10 PCs and not 30 as described earlier was designed to accommodate the much smaller sample size in this analysis.
